# Ilizarov fixator as salvage procedure after frustrating arthrodesis using intramedullary nailing - is there a chance of consolidation?

**DOI:** 10.1007/s00402-024-05602-0

**Published:** 2024-10-03

**Authors:** Alexis Brinkemper, Raimund H. Lülsdorff, Sebastian Lotzien, Christiane Kruppa, Thomas A. Schildhauer, Charlotte Cibura

**Affiliations:** 1 Department of General and Trauma Surgery, BG University Hospital Bergmannsheil, Ruhr-University Bochum, Bochum, Germany; 2 Bürkle-de-la-Camp-Platz 1, 44789 Bochum, Germany

**Keywords:** Retrospective study, Ankle arthrodesis, Ilizarov fixator, Intramedullary nailing, Limb salvage

## Abstract

**Introduction:**

Arthrodesis of the tibiotalar and subtalar joints is a salvage procedure that has been used successfully for years. Treatment options include internal procedures and external procedures. Retrograde intramedullary nailing is considered a safe procedure with a high degree of stability and comfort. Nevertheless, there are cases in which this internal arthrodesis fails and another procedure must be considered. Ilizarov fixator treatment could be a solution for those patients in whom intramedullary nailing has failed. Even if it means another surgical revision - is it possible to finally achieve consolidation with this method?

**Materials and methods:**

In this single-center, retrospective study all documents of patients who underwent tibiotalar and subtalar joints fusion using the Ilizarov external fixator at our institution from 2003 to 2023 as secondary treatment after frustrated first arthrodesis using an intramedullary nail were reviewed. Nineteen patients (17 men and 2 women), with an average age of 55.7 (standard deviation (SD) 8.7, range 34–75) years were included.

**Results:**

On average, 1.7 (SD 1.3, range 1–6) arthrodesis attempt were performed before final Ilizarov fixator arthrodesis. The average time spent in the Ilizarov fixator was 19 (SD 4, range 14–29) weeks. In seven cases (36.8%), both the tibiotalar and subtalar joints received bony consolidation in the end.

**Conclusion:**

If patients have undergone fusion of the tibiotalar and subtalar joints with a retrograde nail and this fails, it is difficult to achieve complete consolidation in the further course. A further attempt at arthrodesis using an Ilizarov fixator is possible, but the overall results are also poor. This procedure must therefore be seen as a last resort before amputation.

## Introduction

Arthrodesis of the ankle joint is a salvage procedure that has been used successfully for years. The most common reasons for arthrodesis are primary osteoarthritis and post-traumatic osteoarthritis after all conservative options have been exhausted [1]. Other causes include joint empyema, rheumatoid arthritis, arthrosis of other origins, charcot arthropathy, chronic joint infections, malpositions and deformities due to paralytic or spastic paralysis and post-traumatic osteonecrosis [2]. Treatment options include internal procedures using plates, screws, nails or arthroscopic fusion and external procedures using external fixators [3, 4]. The objective of this treatment is to achieve the greatest possible reduction of pain, together with the regaining of stability and security. Undesirable but known complications are the lack of fusion, infections, nerve damage and increasing deterioration of adjacent joints [1]. Retrograde intramedullary nailing is considered a safe procedure with a high degree of stability and comfort [5]. In this surgical method, a careful, extensive resection of the cartilaginous joint surfaces and sclerosis zones of the tibiotalar (TT) and subtalar (ST) joint is carried out first, then all potential malalignments are removed and, if necessary, bone defects are filled with autogenous or allogenic bone graft. An intramedullary nail is subsequently inserted in a retrograde fashion through the plantar surface of the hindfoot into the physiological load-bearing axis for a tibiotalocalcaneal (TTC) arthrodesis. A pain-free, intact and radiologically inconspicuous ST joint is a contraindication for this operation [1, 6]. Nevertheless, there are cases in which this internal arthrodesis fails and another procedure must be considered. Due to the amount of bony defect with this technique, we suspect that healing will be difficult to achieve during the course. The Ilizarov ring fixator is an external system that provides dynamic axial fixation with high stability by introducing transfixing wires and screws [3]. It has been described in particular for patients complicated with acute or chronic infections, soft tissue defects, axial malpositions and relevant comorbidities such as diabetes mellitus (DM) or polyneuropathy (PNP) [7–9]. Some authors consider the Ilizarov method to be the “gold standard” in these difficult situations [10]. Ilizarov fixator treatment could also be a solution for those patients in whom intramedullary nailing has failed. Is it still possible to achieve consolidation with this treatment or is amputation the only remaining option? Currently, to the authors’ knowledge, there is no study that has looked at how patients with failed nail arthrodesis have fared after a switch to Ilizarov fixator. Therefore, the aim of this study was to assess the outcomes of patients with failed intramedullary nailing of the ankle who have received a definitive treatment via an Ilizarov fixator.

## Materials and methods

The present study was performed in accordance with the Declaration of Helsinki. Ethical permission for this study was obtained from the local ethics committee. In this single-center, retrospective study all patients who underwent fusion of the TT and ST using the Ilizarov external fixator at our institution from 2003 to 2023 as secondary treatment after frustrated arthrodesis using an intramedullary nail were retrospectively reviewed. To capture all patients with these criteria, a keyword analysis of all digitized files was performed by the authors. The medical records of these patients were reviewed for the following factors: sex, age, associated relevant concomitant diseases, reason for arthrodesis, number of arthrodesis attempts, time spent in the fixator, complications and bony consolidation assessed on the basis of computed tomography (CT) or X-ray images by two of the authors (Table 1). The data were collected anonymously using Microsoft Excel © 2016. The exclusion criteria were as follow: (1) patients without arthrodesis attempts using intramedullary nailing, (2) patients with incomplete records. Our research revealed 21 patients, two deceased before the Ilizarov fixator was removed due to other causes. Our collective therefore consisted of a total of 19 patients (17 men and 2 women), with an average age of 55.7 (standard deviation (SD) 8.7, range 34–75) years. Indications for the first arthrodesis were post-traumatic osteoarthritis with and without instability/foot malalignment in eleven cases (57.9%), acute or chronic infection in five cases (26.3%), primary arthrosis in two cases (10.5%) and charcot arthropathy in one case (5.3%). Concomitant diseases were found in 13 patients (68.4%), of whom six (31.6%) suffered from relevant diseases such as DM and/or PNP. Another risk factor was nicotine abuse in five patients (26.2%) (Table 1). In all 19 cases, primary arthrodesis was performed at another hospital. In all cases, arthrodesis using a retrograde nail was frustrating. When the patients were admitted to our clinic, they had either a non-union or an infected non-union.

**Table 1:** Demographic data and outcome of 19 patients

Patient	Sex	Age	Indication for Ilizarov re-arthrodesis	Duration in frame (weeks)	Complications (major)	Arthrodesis attemps prior to final Ilizarov-fixator	Follow-up (weeks)	Achieved union	Comorbidity
1	M	50	infected non-union	15	pin infection with soft tissue defect	1	29	TT consolidated, ST consolidated	HTN, Nicotine
2	M	41	infected non-union	17		2	32	TT non-union, ST non-union	DM, PNP, Nicotine
3	M	65	infected non-union	15	pin fracture of the two proximal rings	1	1	TT consolidated, ST consolidated	DM, PNP
4	M	49	non-union	16	internal rotation malposition	4	7	TT consolidated, ST consolidated before IF	Nicotine
5	M	60	Subsequent ST arthrosis	17		1	216	TT consolidated before IF, ST consolidated	
6	M	56	infected non-union	25		1	72	TT non-union, ST not adressed in surgery - partially consolidated	HTN, Obesity, Nicotine
7	M	56	non-union	18		1	8	TT non-union, ST non-union	PNP, Nicotine
8	M	56	non-union	19		2	0	TT partially consolidated, ST non-union	DM, PNP, HTN
9	M	58	infected non-union	19		1	17	TT consolidated, ST non-union	DM, HTN, Obesity
10	M	52	non-union	21		3	16	TT consolidated, ST consolidated before IF	
11	M	52	non-union	19	broken forefoot pin	1	24	TT non-union, ST non-union	HTN
12	M	61	infected non-union	14		1	5	TT partially consolidated, ST non-union	
13	M	34	infected non-union	24		2	14	TC non-union	Schizophrenia
14	M	60	non-union	17		6	0	TT consolidated before IF, ST non-union	
15	M	57	non-union	23		2	85	TT consolidated, ST consolidated	Obesity
16	F	58	non-union	15		1	55	TT consolidated before IF, ST partially consolidated	DM, PNP, COPD
17	M	75	Subsequent ST arthrosis	17		1	21	TT consolidated, ST partially consolidated	
18	M	56	non-union	29		1	14	TT consolidated, ST consolidated	HTN
19	F	62	non-union	14		1	19	TT partially consolidated, ST partially consolidated	

The study included a total of 19 patients (17 men and 2 women), with an average age of 55.7 (SD 8.7, range 34–75) years

F female, M male, TT tibiotalar joint, ST subtalar joint, TC tibiocalcaneal, COPD chronic obstructive pulmonary disease, DM diabetes mellitus, PNP polyneuropathy, HTN hypertension

As part of the surgical treatment, implant removal of the nail was initially carried out in all patients. Twelve patients (63.2%) underwent resection at the level of the former TT joint, including resection of the malleolus medialis et lateralis through a medial and lateral approach, removal of the remaining cartilage in the ST joint, and extensive debridement (all infected bones and soft tissues). In three cases (15.3%) only a resection at the level of the former TT joint was performed (in one the lower ankle was not adducted and in the other it was already fused) and in three other cases (15.3%) only the ST joint was debrided and decartilaged because bony bridges already existed in the TT joint. In one other case (5.3%) the entire talus had to be removed resulting in a tibiocalcaneal (TC) arthrodesis. In six cases (31.6%) AO fixator was used first as a staged procedure, because of large soft tissue defects. After successful soft tissue conditioning, the AO fixator was removed, and the Ilizarov fixator was attached.

The Ilizarov frame consisted of three to four rings that were fixed by means of four half pins and two crossed strained 1,8 mm steel olive wires in the talus, two olive wires in the calcaneus and wires for fixing the metatarsus. The wires were clamped up to 110 kg (1080 N) using the tensioning device. The ring system enabled compression of both the TT and the ST joint. In all cases, it was preassembled preoperatively and applied en bloc. In one case, a TC arthrodesis had to be performed after removal of the talus. In this case, the crossed wires were inserted only into the calcaneus and the forefoot was fixed.

**Fig. 1:**
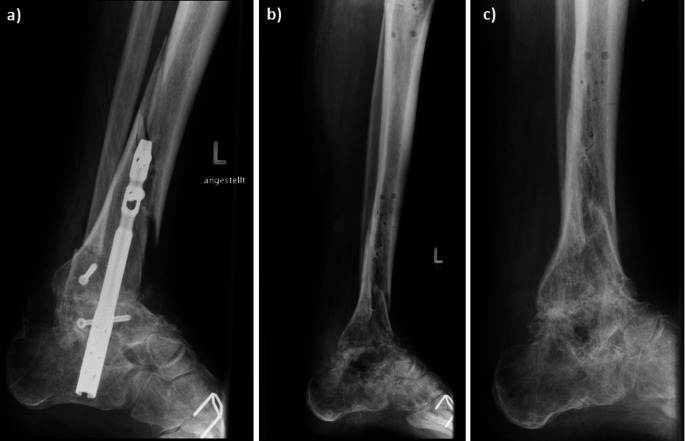
Patient with an infectious situation with persistent non-union and additional tibia fracture. (**a**) Image seven days before the change from nail to Ilizarov fixator. (**b**) Image at day of Ilizarov fixator removal. (**c**) Follow-up three months after removing of the Ilizarov fixator

In eleven patients (57.9%), autologous bone graft was used to fill osseous defects, in two patients (10.5%) a mixture of allogenic and autologous bone graft was used. In six patients (31.6%), none was used. Six patients (31.6%) had an infectious situation in the TT or ST joint. All patients with an infectious situation initially received either a calculated antibiotic treatment or antibiotics in accordance with the resistogram for at least six weeks. Time between first arthrodesis and final arthrodesis was 18 (SD 19, range 1–84) months. After removal of the Ilizarov fixator, the average clinical/radiographic follow-up period was 33 (SD 50, range 0–216) weeks. Figure 1 shows an example of the progression from the lack of union with nail to the follow-up 3 months after removal of the Ilizarov fixator.

## Results

**Fig. 2:**
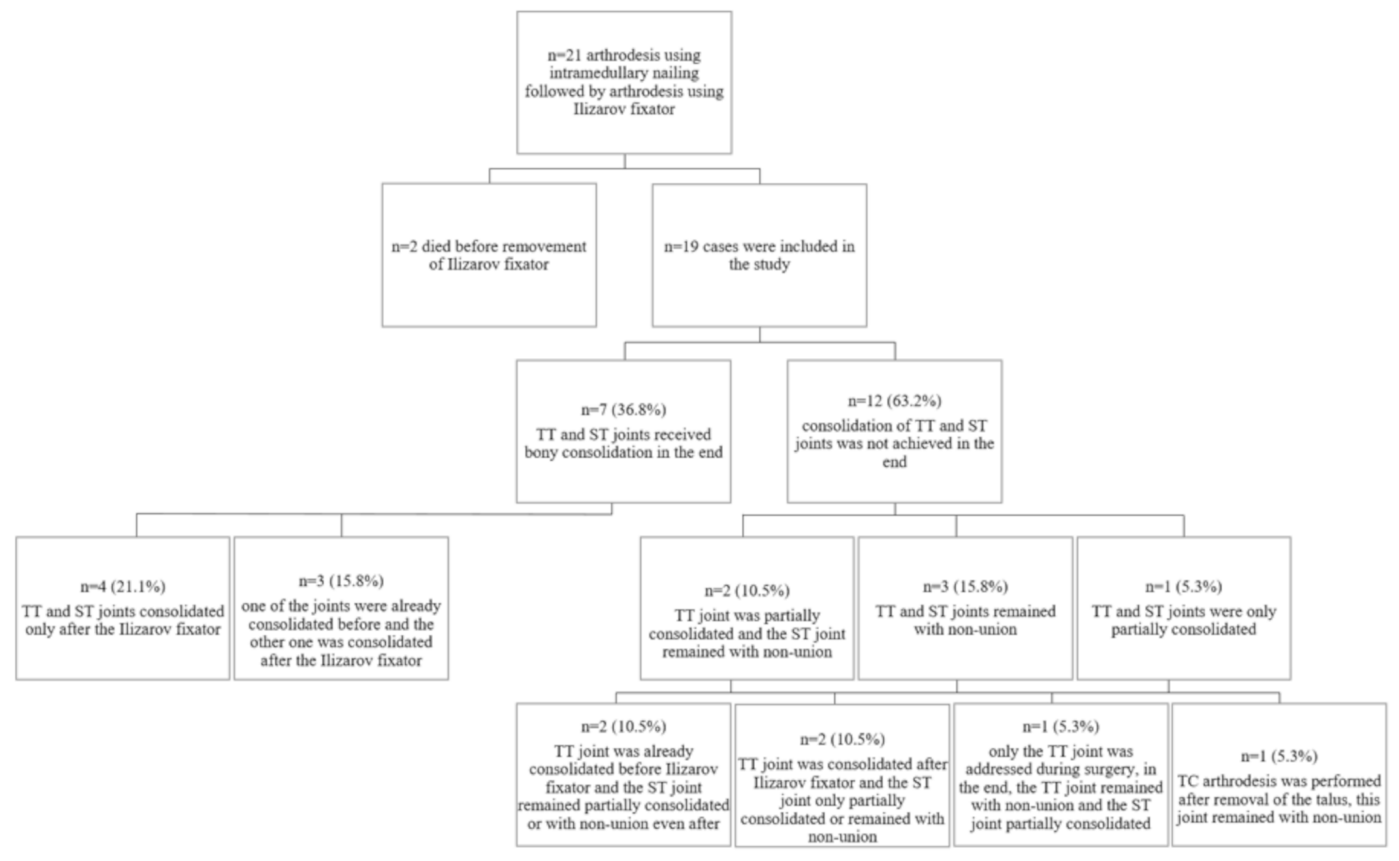
Overview of inclusion and progression of the cases

The average time spent in the Ilizarov fixator was 19 (SD 4, range 14–29) weeks. In seven cases (36.8%), both the TT and ST joints received bony consolidation in the end. Of these, in four cases (21.1%) both joints fused after the Ilizarov fixator treatment and in three cases (15.8%) one of the joints was already consolidated before and the other one was consolidated after the Ilizarov fixator. In twelve cases (63.2%), consolidation of both the TT and ST joint was not achieved even after the Ilizarov treatment. A detailed list can be found in Fig. 2.Two cases where TT and ST joints remained unfused received a recommendation for a further surgery, one for revision and one for amputation. Both patients declined this and were lost to further follow up. All results can be seen in Table 1.

On average, 1.7 (SD 1.3, range 1–6) arthrodesis attempts were performed before final Ilizarov fixator arthrodesis. In twelve patients, the nail was the first arthrodesis attempt and after its failure, the Ilizarov fixator was used. In the other seven patients, 2–6 arthrodesis attempts were made. In five of these cases, nailing was performed first, followed by one or more attempts using screws, plates, wires or Ilizarov fixator. In two cases, nailing was not the first attempt, in one case an Ilizarov fixator had already been used ex domo and in another case, two previous attempts were described without mentioning the method. In three of the seven cases with multiple arthrodesis attempts, a frustrating attempt using an Ilizarov fixator was already made right before the final arthrodesis in domo. One of these cases finally achieved consolidation in the TT and ST joints, in another one TT joint was already consolidated before Ilizarov fixator and the ST joint remained unfused and the other case is the one where TT and ST joints remained unfused and who received recommendation for amputation and then discontinued treatment with us.

In the follow-up treatment after removal of the Ilizarov fixator, 13 cases (68.4%) received a carbon orthosis in which they were allowed to bear full weight. 4 cases (21.1%) were further treated with an orthopaedic shoe and in two further cases (10.5%) there was no documentation of this. All cases had a plaster cast or a Vacoped boot before the orthosis or orthopaedic shoe until the final fitting.

There were four cases (21.1%) of major complications during the ring fixator wearing period. These were: one pin infection with soft tissue defect, one pin fracture of the two proximal rings, one broken forefoot pin and one internal rotation malposition. All complications were resolved during the course of treatment by surgery so that further treatment could be continued without any problems.

## Discussion

Both intramedullary nailing and treatment using an Ilizarov fixator as arthrodesis procedures are established methods. Like all procedures, these have their advantages and disadvantages and there are well-described pros and cons for both techniques in the literature, depending on the initial situation at the joint, the incidence of infection and other factors [4, 8, 11–13].

However, our study was not about the superiority of one procedure over the other in general. If an arthrodesis fails, the aim is firstly to analyze what caused this and secondly which alternative treatment methods can be considered afterwards. Causes of nonunion include infection, failed or inadequate fixation, inadequate bone preparation, poor bone material and inadequate immobilization after surgery [13]. With regard to our collective, we can only comment little on surgery-specific factors, as the retrograde nail treatment was performed in external hospitals. Nevertheless, in some patients from our group, only the TT joint needed to be addressed initially, but the ST joint was fused along with the retrograde nail. This method injures the still intact ST joint. In this context, Kim et al. state that some surgeons argue in favor of primary preventive TTC arthrodesis due to an increased risk of secondary osteoarthritis of the adjacent joints after isolated TT arthrodesis [14]. Like other authors [1, 6, 13], we advise against this procedure. This is particularly true in view of the fact that additional stiffening of the ST joint is associated with a considerably poorer quality of life [15]. In addition, in some cases the ST joint was not completely decartilaged, which makes consolidation difficult and may be a cause of later problems.

Despite high primary stability [5], another problem with the nail could be a lack of compliance with full loading too early. Comorbidities also play an important role in the chance of a satisfactory follow-up result. In a study on 88 patients with internal ST arthrodesis, Chahal et al. showed that smokers and patients with DM had a 3.8- and 18.7-fold higher probability of nonunion and that the worst functional outcome was observed in patients with DM [16]. Similarly, Frey et al. reported higher rates of nonunion associated with diabetes, renal insufficiency, smoking and alcohol consumption, among others, and Cobb et al. found in a controlled case study of 22 patients that patients who smoked had a 14-fold higher rate of nonunion than former smokers and non-smokers [17, 18]. Confirming this association, 13 out of 19 patients included in our study showed comorbidities, including DM, PNP and smoking.

If arthrodesis fails, amputation below the knee is often mentioned as a solution. Some authors, however, present techniques such as a blade T-plate in the form of revision surgery to preserve the limb [19]. Others tend to favor the use of external fixators, as this avoids the use of foreign material at the fusion site, which is especially advantageous in the case of a chronic recurring infection [9, 12, 20]. Supporter of the Ilizarov fixator argue that this procedure limits soft tissue trauma and, as a ring fixator does not usually require a plaster cast, swelling and ulcer recurrence can be better monitored [11, 21]. In addition, full weight bearing can be carried out in the fixator. On the other hand, there are high incidence of pin tract infection, risk of tibial fracture and the need for a second surgery for material removal [11].

All patients in our study received an Ilizarov fixator after the attempt of nailing and, in some cases, further attempts of arthrodesis failed. The time spent in the fixator in our patient population is comparable to other studies using Ilizarov fixators and thus shows no particularity compared to collectives in which the fixator was the first procedure [3, 5, 22].

There are a few studies in which external fixator was used as a salvage procedure after previously unsuccessful arthrodesis of TT or ST joints or after TC arthrodesis. In their study, Easley et al. report on 19 of 22 patients with achieved fusion following revision TT arthrodesis using an external ring fixator after previous nonunion of an internal TT arthrodesis with screws [23]. In two of three further patients in this study in whom a re-arthrodesis with repeated internal procedures using screws was carried out and failed again, fusion was achieved after re-revision with a ring fixator. Midis and Conti had ten revision arthrodesis for patients with aseptic nonunion of the TT joint using the technique of unilateral external fixation [24]. Nine of the ten revision cases had previously undergone internal fixation with partially threaded cancellous screws and one case with an intramedullary rod. Fusion occurred in all ten of their patients. Johnson et al. described six patients, four of whom had failed ankle arthrodesis with infected pseudarthrosis [25]. In three of the four failed infected ankle fusions, a successful TC fusion was achieved. Hawkins et al. reported 21 cases of complex pathologies of the distal tibia or failed ankle arthrodesis that were treated with the Ilizarov fixator [26]. Of the 20 cases available for evaluation, 16 achieved good results with solid arthrodesis of the ankle. Katsenis et al. reviewed the results of twenty-one patients who received revision of a failed fusion with use of the Ilizarov fixator [21]. Eight patients had TT joint fusion, eleven had TT and ST joint fusion, and two had pantalar fusion. Union was achieved in all ankles accompanied by excellent functional result in fifteen patients, good in three, fair in two, and poor in one.

However, in most of these studies, the method of initial arthrodesis is not described or a method other than intramedullary nailing was used. In our series, we observed a satisfactory result in slightly more than one third of patients. One reason for this poor result may be the lack of experience with this rare situation as we only had 19 cases in the past 20 years. Yet, it must be taken into account that we are dealing with a difficult patient population, some of whom with a long medical history and many previous operations. Due to the massive bone loss caused by the previous arthrodesis with nailing and the coexisting comorbidities, a better result is unfortunately not always achievable. In view of the threat of amputation, the authors believe that in some cases it is well worth the effort.

After the fixator is removed, the question arises as to whether further treatment, for example with an orthopaedic shoe or orthosis, is indicated. In this context, Cibura et al. showed that treatment with a carbon orthosis can lead to further consolidation in a difficult patient population [7]. In line with this, from 13 cases of our population who received a carbon orthosis after removal of the Ilizarov fixator, seven (53.9%) were fully consolidated. In three others (together 76.9%) one joint was consolidated and the other partially consolidated or remained unfused.

This study had several limitations. First, the number of 19 cases was small, and the study had a retrospective design. Another limitation is the inconsistent follow-up imaging. In some cases CT images were available, in others only radiographs. In addition, comparable studies are not available. However, the results presented here show that revision surgery using an Ilizarov fixator potentially offers the chance to improve the situation and thus counteract a possible amputation, but is not a patent solution for this patient group. Our hypothesis was that the bone defect caused by intramedullary nailing is a major reason for difficult re-arthrodesis. Based on the data available here, we consider this hypothesis to be confirmed, even if there are positive exceptions. From the authors’ point of view, treatment with an Ilizarov fixator after failed nail arthrodesis remains a salvage procedure overall and represents the last resort before amputation.

## Conclusion

If patients have undergone fusion of the TT and ST joints with a retrograde nail and this fails, it is difficult to achieve complete consolidation in the further course. A further attempt at arthrodesis using an Ilizarov fixator is possible, but the overall results are also poor. This procedure must therefore be seen as a last resort before amputation.

## Data Availability

The authors confirm that the data supporting the findings of this study are available within the article or its supplementary materials.

## Abbreviations

TT: Tibiotalar
ST: Subtalar
TC: Tibiocalcaneal
TTC: Tibiotalocalcaneal
DM: Diabetes mellitus
PNP: Polyneuropathy
CT: Computed tomography
SD: Standard deviation
F: Female
M: Male
COPD: Chronic obstructive pulmonary disease
HTN: Hypertension
